# Editorial: Iron deficiency and excess: diagnosis, management and impact on human health

**DOI:** 10.3389/fnut.2025.1683999

**Published:** 2025-09-30

**Authors:** Patrícia Molz, Piotr F. Czempik, Gomathi Ramaswamy

**Affiliations:** ^1^Graduate Program in Biosciences, Federal University of Health Sciences of Porto Alegre, Porto Alegre, Brazil; ^2^Department of Anesthesiology and Intensive Care, Faculty of Medical Sciences in Katowice, Medical University of Silesia, Katowice, Poland; ^3^Department of Community Medicine and Family Medicine, All India Institute of Medical Sciences, Bibinagar, India

**Keywords:** diet, iron, deficience, overload, micronutrient

## Introduction

This editorial summarizes the main findings published within the scope of the Research Topic *Iron deficiency and excess: diagnosis, management and impact on human health*, available in Frontiers in Nutrition. The selected articles address recent advances in understanding, diagnosing, and treating iron-related disorders, with a focus on vulnerable populations and innovative therapeutic strategies.

Iron is an essential micronutrient that plays a pivotal role in numerous physiological processes, such as oxygen transport via hemoglobin, nucleic acid and protein synthesis, and energy production through oxidative phosphorylation. Disruptions in iron homeostasis—manifesting as either iron deficiency (ID) or iron overload—represent two extremes of the metabolic spectrum, each with significant implications for human health. Iron deficiency is one of the most widespread nutritional disorders globally and is primarily associated with iron-deficiency anemia (IDA). Clinical manifestations of IDA include fatigue, dizziness, dyspnea, tachycardia, and, in chronic cases, heart failure. In addition to anemia, iron deficiency contributes to a range of adverse outcomes, such as headaches in adults, cognitive and developmental delays in children, pregnancy-related complications, and an increased susceptibility to infections due to impaired innate and adaptive immunity. Conversely, iron overload can induce oxidative stress and organ dysfunction, primarily affecting the liver, heart, and pancreas. This can lead to conditions such as diabetes mellitus, infertility, and thyroid dysfunction. In recent decades, advances in diagnostic techniques and therapeutic strategies have significantly shaped the management of iron-related disorders. This editorial condenses the findings from nine recent scientific articles, highlighting progress in understanding, diagnosing, and treating iron deficiency and overload.

## Iron deficiency: global burden and vulnerable populations

Iron deficiency disproportionately affects vulnerable groups, including women of reproductive age, children, and the elderly. Zhang et al. reported that despite a global decline in nutritional deficiencies between 1990 and 2021, ID continues to affect women and low-income populations, particularly in countries with low social development indices. In India, Hashmi and Singh documented a concerning rise in anemia among Muslim women—from 48.8% to 55.6% over 23 years—with higher prevalence observed among adolescents, rural residents, and individuals with limited education. Similarly, Wang et al. identified dietary iron deficiency as the predominant cause of anemia in elderly Chinese populations, especially in rural and underdeveloped regions.

## Iron overload: emerging associations and risks

Recent studies have expanded the clinical spectrum of iron overload, linking it to conditions such as fecal incontinence (FI) and human papillomavirus (HPV) infection. Li et al., using NHANES data from 2007 to 2010, identified an inverted U-shaped relationship between iron intake and FI, and a negative association between serum iron levels and solid FI, particularly in elderly women—suggesting that both iron deficiency and excess may impair gastrointestinal function. Chen et al. reported an L-shaped association between iron intake and HPV infection, with adequate iron levels reducing the risk of infection by 23.2%. In Ethiopia, Endrias et al. found a 60.93% prevalence of anemia among chronic kidney disease patients, a condition exacerbated by comorbid diabetes and advanced disease stages.

## Innovations in iron deficiency treatment

Several novel and cost-effective interventions have been proposed to address iron deficiency. For example, Elmrayed et al. demonstrated that fortified infant cereals significantly reduced anemia prevalence by 32% among Egyptian children, while also lowering public health expenditures—underscoring their potential as a scalable intervention. Huang et al. introduced a new iron supplement, iron-chelated leek skin peptides (PSP-Fe), which not only restored hematological parameters more effectively than ferrous sulfate in anemic rats but also positively influenced gut microbiota composition. Additionally, Wagiu Basrowi et al. explored the regulatory role of microRNAs (miR-15a, miR-24) in erythropoiesis, proposing protein-based therapies to modulate their expression as a promising avenue for IDA treatment.

## Conclusion

Iron is indispensable for human health, and maintaining its homeostasis is crucial to preventing the deleterious effects of both deficiency and overload. Iron deficiency remains a pressing global health concern, particularly among high-risk populations. Current research emphasizes the importance of early screening, continuous clinical monitoring, and implementing cost-effective, nutritionally adequate, and culturally tailored interventions. Continued scientific inquiry and innovation are essential to mitigating the burden of iron-related disorders and improving population health outcomes.

